# Hemoglobin-Based Oxygen Carriers: Selected Advances and Challenges in the Design of Safe Oxygen Therapeutics (A Focused Review)

**DOI:** 10.3390/ijms26199775

**Published:** 2025-10-08

**Authors:** Waldemar Grzegorzewski, Anna Czerniecka-Kubicka, Katarzyna Gołda, Alicja Niedźwiedzka, Hanna Wollocko, Michał S. Majewski, Joanna Wojtkiewicz

**Affiliations:** 1 Faculty of Biology, Nature Protection, and Sustainable Development, University of Rzeszow, 35-310 Rzeszow, Poland; kasiagolda278@gmail.com; 2 Faculty of Medicine, Department of Pharmaceutical Technology and Medical Physics, University of Rzeszow, 35-959 Rzeszow, Poland; 3 Independent Researcher, 02-777 Warsaw, Poland; alicjadniedzwiedzka@gmail.com; 4 Department of Basic and Clinical Sciences, TOURO College of Osteopathic Medicine, Middletown, NY 10940, USA; hannaw@oxyvita.us; 5 OXYVITA Inc., Middletown, NY 10940, USA; 6 Department of Pharmacology and Toxicology, Faculty of Medicine, University of Warmia and Mazury, 10-082 Olsztyn, Poland; michal.majewski@uwm.edu.pl; 7 Department of Physiology and Pathophysiology, Faculty of Medicine, Collegium Medicum, University of Warmia and Mazury, 10-082 Olsztyn, Poland; joanna.wojtkiewicz@uwm.edu.pl

**Keywords:** blood substitute, hemoglobin, oxygen carrier

## Abstract

Blood transfusion is a routine yet resource-intensive medical procedure. Increasing global demand, limited donor availability, and logistical and ethical constraints have driven the search for adequate blood substitutes. Hemoglobin-based oxygen carriers (HBOCs) represent a promising class of therapeutics designed to mimic the oxygen transport function of red blood cells while overcoming the challenges of storage, compatibility, and infection risk. Despite decades of research, no HBOC has yet met all criteria for widespread clinical use. This review summarizes recent advances in the design and development of hemoglobin derivatives, with a focus on their biochemical properties, safety profiles, and oxygen delivery capabilities. We also discuss current limitations and translational barriers. The successful implementation of HBOCs could significantly improve transfusion strategies, especially in emergency medicine, military applications, and resource-limited settings. Continued innovation is essential to bring safe and effective oxygen therapeutics into routine clinical practice.

## 1. Introduction

Hemoglobin (Hb), a key component of blood, plays a fundamental role in biology, chemistry, and medicine due to its unique structural and functional properties. As a bidirectional respiratory carrier, hemoglobin is responsible for transporting oxygen from the lungs to peripheral tissues and facilitating the return transport of carbon dioxide. In arterial blood, it exhibits high affinity for oxygen and low affinity for carbon dioxide, hydrogen ions, organic phosphates, and chloride ions. Disorders affecting the quantity or structure of hemoglobin can result in severe physiological dysfunctions. Additionally, the clinical use of donated blood is limited by frequent shortages, strict storage requirements, and a relatively short shelf life [1]. There are also persistent concerns about the potential transmission of blood-borne pathogens such as HIV, hepatitis B virus (HBV), and hepatitis C virus (HCV), despite advances in donor screening and viral inactivation procedures [2]. These challenges have driven interest in the development of hemoglobin-based oxygen carriers (HBOCs) as potential blood substitutes [3]. This review summarizes the current progress in the design and application of HBOCs. The literature was selected through a structured search of Google Scholar and PubMed databases using the keywords “hemoglobin,” “oxygen carriers,” and “hemoglobin-based.” The review includes original research articles, reviews, and clinical studies published in the English language.

**Figure 1:**
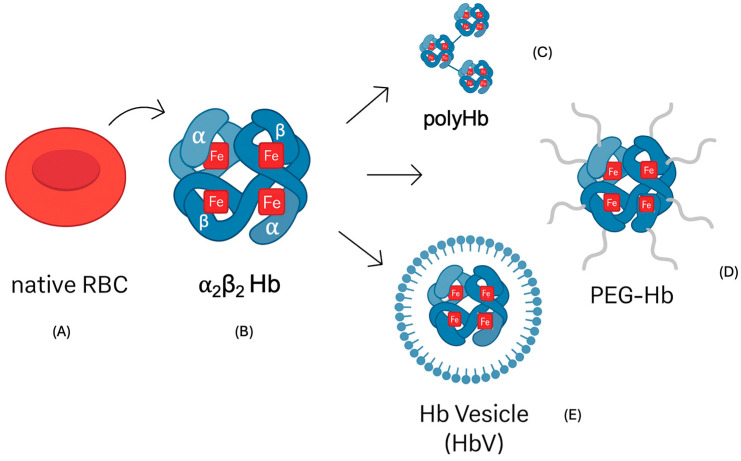
Hemoglobin-based oxygen carriers (HBOCs) compared with native red blood cells. (**A**) Human erythrocyte. (**B**) Tetrameric hemoglobin (α_2_β_2_). (**C**) Polymerized Hb. (**D**) PEGylated Hb. (**E**) Hb vesicle (encapsulated Hb). Figure 1 was generated using images provided by Servier Medical Art (Servier; https://smart.servier.com/, accessed on 30 September 2025), licensed under a Creative Commons Attribution 4.0 International License, with additional editing using Canva AI (accessed on 30 September 2025).

Oxygen is a crucial molecule involved in gas exchange in vertebrate erythrocytes. Hemoglobin functions as an α_2_β_2_ tetramer within RBCs, ensuring efficient oxygen transport. The development of hemoglobin-based oxygen carriers (HBOCs) has therefore attracted significant attention as potential alternatives to donor red blood cells (Figure 1).

## 2. Characteristics of Hemoglobin

Hemoglobin is a respiratory pigment that is responsible for the red color of blood. It is found in erythrocytes, where it can form crystalloids [4]. The concentration of hemoglobin in one cell is 340 g/L [5]. The hemoglobin molecule consists of four polypeptide chains: two alpha globin protein chains composed of 141 amino acids and two beta globin chains composed of 146 amino acids each. These chains are similar in structure and size. The α and β subunits are composed of 7 and 8 helices, respectively, named A to H, which are connected by nonhelical segments. Helices E and F form a pocket that binds the heme group. A crucial element, iron, is anchored in the proximal pocket of the heme by the imidazole of the histidine residue located on helix F. This arrangement enables iron to bind molecular oxygen and other gases, stabilizing them [6]. In addition to the protein components, the hemoglobin molecule also includes four heme groups. The coordination center of each heme contains an iron atom in its second oxidation state.

Types of Hemoglobin

There are four main types of hemoglobin:

- HbA—the basic hemoglobin found in adults, composed of two alpha chains and two beta chains,
- HbF—fetal hemoglobin, present in infants, composed of two alpha chains and two gamma chains,
- HbA2—minor form of hemoglobin present only in 2.5% of the human population in adults, composed of two alpha chains and two delta chains,
- HbS—hemoglobin of sickled red blood cells, composed of two alpha chains and two mutated beta chains.

During human development, the subunit composition of the hemoglobin tetramer changes. The γ chain, present in fetal hemoglobin (HbF), is gradually replaced by a β chain around 90 days after birth. While the β subunit chain is synthesized during pregnancy, the transition to the adult hemoglobin tetramer (HbA) is completed only a few weeks after birth [7].

## 3. Oxygen and Carbon Dioxide Transport by Hemoglobin

Each heme group in hemoglobin can bind one oxygen molecule, allowing a single hemoglobin molecule to carry up to four oxygen molecules [6]. This binding occurs through the interaction of oxygen with the iron atom in heme, without changing the iron’s oxidation state [6]. The binding of the first oxygen molecule in the lungs facilitates the attachment of additional oxygen molecules (cooperative binding). Similarly, the release of one oxygen molecule in the tissues promotes the release of the remaining molecules [8]. Deoxyhemoglobin (deoxyHb), the reduced form of hemoglobin, binds oxygen in pulmonary capillaries and becomes the oxygenated form, oxyhemoglobin (oxyHb). Although oxyHb is generally stable, it can be oxidized under certain conditions that convert Fe(II) in the heme molecule to Fe(III), forming methemoglobin, which cannot carry oxygen [6]. Hemoglobin exists in two conformational states: the tensed T state, associated with low oxygen affinity, and the relaxed R state, associated with high oxygen affinity. Hemoglobin molecules are in equilibrium between these states, and oxygen binding shifts the equilibrium toward the R state. This underlies cooperative binding, as successive oxygen molecules increasingly stabilize the R conformation [8]. Structural rearrangements accompanying the T to R transition include the movement of the iron atom into the plane of the heme and 15° rotation of the α_2_/β_2_ dimer relative to the α_1_/β_1_ dimer, completing the conformational change.

The measure of the affinity of various hemoglobins for oxygen, called P50, is the partial pressure of oxygen at which half of the hemoglobin is saturated with oxygen [9]. This corresponds to the molecular pressure of oxygen at which the oxygen saturation of hemoglobin is 50%. The total oxygen content in the blood consists of dissolved oxygen in the plasma and oxygen bound to hemoglobin. Under normal pulmonary conditions, approximately 97–98% of oxygen in the blood is bound to hemoglobin in red blood cells, and less than 2% is dissolved in the plasma [10].

The hemoglobin dissociation curve has a sigmoidal shape; therefore, when the partial pressure of oxygen exceeds 50 mmHg, the hemoglobin oxygen saturation is almost 90%, which is a form of protection for the body [10]. In human blood, under physiological conditions (pH 7, temperature 37 °C), the partial pressure of oxygen at which 50% of hemoglobin is saturated (P50) is approximately 26 mmHg [9]. In the lungs, hemoglobin in red blood cells is ~97% saturated with oxygen. In systemic venous blood at rest, saturation is typically ~70–75% and can fall further in highly metabolic or hypoxic tissues [9,10]. The allosteric balance of hemoglobin is controlled by effectors that shift the balance toward the T state or the R state. These allosteric effectors bind at sites other than heme and modulate hemoglobin’s affinity for oxygen. The balance between the T and R states is influenced by endogenous heterotropic ligands such as 2,3-bisphosphoglycerate, hydrogen ions, chloride ions, and carbon dioxide, as well as by synthetic allosteric effectors [9].

Carbon dioxide produced during cellular respiration is also transported by hemoglobin, primarily in the form of bicarbonate ions. These ions are removed from the red blood cell cytoplasm through the chloride shift, when bicarbonate ions are exchanged for chloride ions [5].

Hemoglobin also functions as a buffer by binding with hydrogen ions, thereby protecting against dangerous pH changes. In red blood cells, approximately 12% of carbon dioxide also combines with hemoglobin via chains of terminal amino groups, creating carbaminohemoglobin. The accumulation of CO_2_ and hydrogen ions lowers hemoglobin’s affinity for oxygen, a phenomenon known as the Bohr effect, which facilitates oxygen unloading in metabolically active tissues [5,9]. In the lungs, this process is reversed: oxygen binds to deoxyHb and hydrogen ions are released, and these recombine with bicarbonate to form carbonic acid. Under the influence of carbonic anhydrase, carbonic acid dissociates into water and carbon dioxide, the latter diffusing from the plasma to the alveoli for exhalation [5].

## 4. Hemoglobin-Based Oxygen Carriers

Hemoglobin-based oxygen-carrying preparations are semisynthetic products that use various forms of hemoglobin as oxygen carriers [11]. These include natural hemoglobin, genetically modified hemoglobin, hemoglobin enclosed in microparticles, conjugated hemoglobin, or hemoglobin cross–linked and polymerized. Hemoglobin is typically derived from bovine erythrocytes or obsolete human erythrocytes, possibly from recombinant sources. These preparations were developed to provide an alternative to blood transfusions and as oxygen therapeutics in ischemic conditions [12].

### 4.1. Unmodified Hemoglobin

Initially, natural hemoglobin was investigated as an oxygen carrier, but this approach did not yield the expected results [12]. The presence of free hemoglobin in plasma creates several problems. Its circulation time is short because hemoglobin tetramers dissociate into αβ dimers, which rapidly bind to haptoglobin and are cleared by macrophages in the liver and spleen via the CD163 receptor pathway [13,14]. The hemoglobin-haptoglobin complex is internalized and degraded in lysosomes, preventing renal filtration and conserving iron. However, the binding capacity of haptoglobin is limited, and once it is saturated, excess dimers are filtered by the renal glomeruli, leading to hemoglobinuria and potential kidney damage [13,14]. Free hemoglobin can also extravasate through the vascular endothelium due to its small size and scavenge nitric oxide, causing vasoconstriction and increases in systemic mean arterial pressure [15,16]. In addition, because free hemoglobin lacks interaction with 2,3-bisphosphoglycerate, its oxygen affinity is abnormally high, which impairs oxygen release to tissues [17]. These toxic effects, including oxidative stress, vascular dysfunction, and renal injury, limited the clinical use of unmodified hemoglobin as a blood substitute [15,16,18].

### 4.2. Molecular Modifications of Hemoglobin

Due to the negative effects of the presence of hemoglobin dimers in plasma, research shifted toward stabilizing the tetrameric form of hemoglobin by modifying the molecule and attempting to encapsulate hemoglobin [19,20]

The polymerization (poly) of hemoglobin molecules increases the size of cell–free hemoglobin, thereby minimizing extravasation and extending its half–life in the intravascular circulation. This process is typically carried out using agents such as glutaraldehyde and glycolaldehyde. A key challenge is controlling the molecular weight of the polymer and ensuring sterile purification of the product [19,21]. Each PolyHb molecule consists of several hemoglobin molecules, and various types have developed over time, divided into generations. One such preparation, based on the polymerization of bovine hemoglobin with glutaraldehyde (HBOC-201, Hemopure), has been approved in South Africa for the treatment of adult patients with acute anemia to delay the need for red blood cell transfusions. This product has also been made available under expanded-access or compassionate-use programs in the United States, particularly in patients who decline allogeneic transfusions for religious reasons, such as Jehovah’s Witnesses [12]. In contrast, in Europe and the United States, only one oxygen-carrying hemoglobin-based blood substitute has been approved to date by the Food and Drug Administration (FDA) for veterinary use only [22,23]. This preparation consisted of purified bovine hemoglobin polymerized with glutaraldehyde embedded in modified Ringer’s lactate. The CVM (Center for Veterinary Medicine/part of FDA) approved this HBOC product in 1998 for the treatment of severe anemia in dogs [22,23]. The product was well received in the veterinary medicine; commercial availability has varied over time [24].

Hemoglobin molecules can be crosslinked between two α chains or two β chains, typically using agents such as raffinose or diaspirin. It is believed that cross–linking of α–α chains may prevent the dissociation of oxyHb into αβ dimers, which are usually readily excreted by the kidneys. Cross–linked tetramers stabilize the molecule, thereby preventing renal filtration [20]. Intramolecular cross–linking between two α-subunits and two β-subunits using site–specific cross-linking of diaspirin further enhances the structural stability of the hemoglobin molecules.

Intramolecular cross–linking of hemoglobin was the first method used to prevent the dissociation of the hemoglobin tetramer by chemically creating covalent cross–links between the two α chains of the tetramer. The first trial candidate was a preparation based on human hemoglobin. To create diaspirin crosslinked hemoglobin (DCLHb, HemAssist), the reagent (3,5-diobromo-salicyl)-fumarate was used to form a covalent bond between two α subunits (Lys99 α1 and Lys99 α2) in the deoxy conformation. In animal testing, this preparation extended circulation time to 12 h, and its P50 value of approximately 30 mmHg was similar to that of natural hemoglobin. The Hill coefficient, however, was reduced to around 1.2, reflecting a marked loss of cooperativity compared to native hemoglobin (*n* = 2.5–3.0). The reported shelf life of DCLHb produced from expired human blood was ~9 months frozen, ~24 h refrigerated [17,19]. Preclinical and clinical settings explored its effects on post-stroke neuroprotection in mice, intraoperative anemia in sheep, or cardiopulmonary resuscitation outcomes in pigs [25,26]. However, intensive preclinical and clinical studies revealed increased mortality in DCLHb-treated patients compared to controls. This was attributed to nitric oxide scavenging by free Hb, which caused vasoconstriction, systemic hypertension, and impaired microvascular perfusion [15,16,18,26]. After further investigation, all products based on cross–linked hemoglobin were eventually terminated [17,18,26].

Conjugation of hemoglobin with antioxidant enzymes has the potential to protect hemoglobin molecules from free radical damage. These conjugated enzymes also increase the overall molecular size of hemoglobin from 3 nm to 15 nm, which reduces renal clearance and limits the ability of hemoglobin to penetrate the walls of blood vessels; thus, it prevents nitric oxide scavenging. Enzymes can also be substituted by small-molecule mimetics. For example, polynitroxylated hemoglobin incorporates nitroxide radicals with superoxide dismutase- and catalase-mimetic activity, enhancing resistance to oxidative stress. Similarly, metalloporphyrins (such as iron or manganese porphyrins) have been investigated as catalytic mimetics of superoxide dismutase and peroxidases, designed to protect hemoglobin from reactive oxygen species-mediated damage [13,17,26]. However, clinical trials of such preparations showed a significantly increased risk of heart attack and death in the study group; therefore, they were withdrawn from clinical development [18,26]. Further attempts to develop chemically modified preparations also failed to meet expectations. Their half-life remained short, approximately 12–18 h, constraining them to short–term use during sudden blood loss [11,26]. Clinical trials revealed serious complications, including oxidative damage linked to iron oxidation, activation of macrophages, release of cytokines, inflammation of blood vessels, hypertension, nephrotoxicity, and organ damage. These adverse effects were largely attributed to the nitric oxide scavenging, which persisted despite increased molecular size, and to oxidative toxicity from heme redox cycling, generating reactive oxygen species. In addition, activation of inflammatory pathways contributed to endothelial dysfunction and organ injury [13,18,26,27].

Beyond these three main strategies of molecular modification of hemoglobin, hybrid approaches have also been explored. These include combinations such as cross–linked and polymerized hemoglobin or cross–linked and conjugated hemoglobin [19]. The main strategies for molecular modification of hemoglobin, their representative products, intended benefits, and limitations are summarized in Table 1.

### 4.3. Clinical Products and Their Outcomes

Earlier generations of HBOC commercial products achieved varying molecular sizes through different techniques of chemical modification of hemoglobin [12]. Examples of these molecular sizes are presented in the table below (Table 2).

The FDA accepted some of the HBOC products into phase I to III clinical trials; however, none of them were approved for therapeutic use. While many of these products demonstrated effectiveness in oxygen delivery during several pre-clinical/clinical trials, side effects resulting from intravenous (i.v.) application, such as extravasation, toxicity of chemical linkers/conjugation agents (e.g., glutaraldehyde), and oxidative and cellular damage, prevented the FDA’s regulatory approval for clinical use [18,26,27,28,29]. A newer HBOC preparation, still in development, is produced under the “zero–link” polymerization process of bovine hemoglobin. This novel modification of and the resulting molecular size of polymerized hemoglobin have significantly advanced preclinical research carried out in recent years. The stability of this preparation stems from its unique molecular structure, obtained as a result of zero-link polymerization, which includes a series of activation steps leading to the formation of pseudopeptide bonds between adjacent tetrameric hemoglobin molecules [28,30,31].

### 4.4. Encapsulation Technologies

Hemoglobin molecules from humans or animals can be enclosed in a two–layer phospholipid capsule, mimicking the cell membrane of red blood cells, through a process called encapsulation. The phospholipid layer with cholesterol molecules acts as a red blood cell membrane, which increases the stiffness and mechanical stability of hemoglobin surrounded by liposomes. Clinical translation efforts are ongoing in Japan, building on preclinical and formulation advances [32,33].

At the end of the 20th century, the “stealth liposome” technology was developed, in which lipid nanoparticles are coated with polyethylene glycol (PEG) to enhance their stability and prevent uptake by macrophages. The aim of this modification was to ensure a longer residence time in the circulation [32]. This technology was applied to prepare hemoglobin vesicles (HbVs), and by coating the particles with PEG, the circulation time increased to approximately 60 h in some animal models [32,34]. Studies have shown that the ability of HbV to transport oxygen is similar to that of natural erythrocytes, with similar oxygen saturation and release kinetics. This is due to the phospholipid layer of liposomes, which resembles the cell membrane of erythrocytes. Enclosing hemoglobin in liposomes also increases the bioavailability of nitric oxide, which reduces the vasoconstrictive effect [32,34]. Liposomes, ranging from 100 to 200 nm in size, are too large to be filtered out by the kidneys, which helps prevent nephrotoxicity [32]. Another advantage of these preparations is that they are free from pathogens and blood group antigens, making them safe for administration to patients. Additionally, liposomes stored in liquid nitrogen can be preserved for two to three years. In recent years, HbV preparations have undergone clinical trials in animal models for use as a blood substitute in transfusions, in the oxygenation of ischemic and transplanted organs, and for alleviating the effects of massive blood loss [32,34]. While research indicates that these preparations are promising, clinical development programs such as TRM-645 have highlighted formulation issues [35]. Current research efforts are focused on overcoming these issues so that these preparations can be used in clinical applications in the future.

Recently, Grzegorzewski et al. (2025) [36] demonstrated that OxyVita^®^C, a zero-link polymerized HBOC, significantly improved viability and reduced acute tubular necrosis in ex vivo preserved rabbit kidneys. This study highlights the translational potential of HBOCs in organ preservation, supporting aerobic metabolism during cold storage and limiting tissue injury compared with conventional anaerobic solutions [36].

The subsequent advancement in hemoglobin-based oxygen carriers involved the development of polymersome-encapsulated hemoglobin (PEH) preparations [37]. Polymerosomes are synthetic polymer vesicles with nanometric dimensions (typically 50–300 nm) that can safely encapsulate significant amounts of hemoglobin. Carrier size influences vascular interactions [38]. These vesicles are composed of synthetic amphiphilic copolymers that confer a range of favorable biological properties, including enhanced chemical and mechanical stability, complete biodegradability, reduced immunogenicity, tissue targeting, and controlled degradation of the carrier [38,39]. The hemoglobin used for this process may be of bovine or human origin. PEH preparations exhibit physiological properties of binding and releasing oxygen that are similar to those of natural red blood cells [37,40]. Additionally, their oncotic pressure closely resembles that of human blood, and their small, uniform size facilitates mass production and ensures prolonged storage stability [37,39].

Enclosing hemoglobin within the core of a polymersome provides a protective barrier, minimizing contact with plasma and surrounding tissues. This configuration also enables fine-tuning of physicochemical properties through the strategic selection of polymer types, as well as control over their molecular weight and concentration. Furthermore, polymersomes can be engineered to be both biocompatible and biodegradable [39]. Compared to liposome-based formulations, PEH exhibits improved in vitro chemical stability, increased bioavailability, and extended half–life [39]. The polymersome membrane is significantly thicker than that of liposomes, offering enhanced mechanical strength and increased intravascular stability. However, this increased membrane thickness may impede the diffusion of small polar gases such as nitric oxide [39,40]. PEH formulations also demonstrate favorable storage properties, maintaining stability in terms of size and morphology for several months [39].

Li and coauthors demonstrated that asymmetric copolymer vesicles can serve as a hemoglobin vector for ischemia therapy in vivo, supporting the translational potential of PEH systems [41]. Further in vitro and in vivo investigations are ongoing to validate these findings and optimize the formulation.

Future research on PEH systems should focus on evaluating sterilization techniques, storage stability, and hemoglobin bioactivity post-sterilization. In addition, therapeutic performance should be systematically assessed in appropriate animal models to establish clinical relevance.

A distinct approach to oxygen carrier development uses naturally occurring extracellular hemoglobins. One example is the giant erythrocruorin from the marine lugworm Arenicola marina (M101, commercialized as HEMO2life^®^). This molecule has a molecular mass of ~3600 kDa, arranged in a hexagonal-bilayer structure, and contains 156 globin chains (plus non-globin linker chains) capable of transporting up to 156 oxygen molecules when fully saturated. Unlike vertebrate hemoglobin, M101 remains stable across a wide range of temperatures and pH values and displays intrinsic antioxidant activity [42,43]. These properties have led to its application as an additive in organ preservation solutions. Preclinical studies demonstrated that M101 improved oxygenation and reduced ischemia–reperfusion injury in kidney, liver, and heart grafts [42,44]. In kidney transplantation, human data suggest a reduced incidence of delayed graft function and a favorable safety profile for M101-supplemented preservation solutions [45]. Research is ongoing to validate these findings in larger controlled trials and in other transplant settings [46,47,48].

### 4.5. Design Considerations and Future Directions

Chemical modifications of hemoglobin enable precise control over the oxygen affinity of HBOCs. The design of P50 remains a critical and debated issue in HBOC development. Earlier-generation HBOCs aimed to replicate the P50 of natural hemoglobin (about 27 mmHg) or maintained slightly higher values—for example, Hemopure (glutaraldehyde cross-linked bovine Hb) and Polyheme (piridoxilated, glutaraldehyde-crosslinked human Hb). In contrast, some newer formulations such as (pegylated human Hb) and OxyVita (zero-link polymerized hemoglobin) exhibit slightly lower P50 values, around 4–6 mmHg [26,28,29,30]. A lower P50 may promote targeted oxygen release in the microcirculation and reduce the generation of reactive oxygen species [8,26,28,29,30].

Interestingly, acellular hemoglobins naturally exist in several terrestrial and marine environments. Species such as *Arenicola marina* and *Lumbricus terrestris* rely on large, stable, extracellular polymeric Hbs to transport oxygen. The hemoglobin of *Lumbricus terrestris* is particularly notable, containing 144 heme groups and a molecular weight of 3.6 MDa [43,44].

Recent HBOC research has shifted toward the development of bioengineered oxygen carriers designed to mimic the structure and function of human hemoglobin. These include synthetic heme proteins like hemerythrin and myoglobin, as well as genetically engineered hemoglobins (GEHs). However, several key challenges remain unresolved, including product toxicity, immunogenicity, molecular stability, and in vivo oxygen delivery efficiency [17,30,43].

## 5. Conclusions

Attempts to create oxygen carriers based on hemoglobin have been ongoing for many years in both academia and the pharmaceutical industry. Several manufacturers have conducted clinical trials, but due to concerns about their safety and efficacy, none of these products have been approved for general use. Moreover, the mechanism of action of the cell-free, chemically modified hemoglobin remains under investigation, which has contributed to the lack of regulatory approval in both Europe and the United States. One of the main obstacles in developing hemoglobin-based oxygen carriers is effectively mimicking the oxygen-carrying ability of hemoglobin. A successful preparation must meet strict criteria for both effectiveness and safety. It must be free from toxins and impurities, capable of transporting oxygen throughout the body and releasing it where needed, universally compatible across blood types, long-lasting, and suitable for extended storage. Although the preparations developed so far have not been approved for general use, ongoing intensive research continues to offer hope for the development of an ideal oxygen carrier in the future.

## Figures and Tables

**Table 1:** Molecular modification strategies for hemoglobin-based oxygen carriers (HBOCs). Own elaboration (authors’ original compilation).

Strategy	Chemical Modification	Example Product(s)	Intended Benefit	Main Limitation(s)	Clinical Status
Polymerization	Glutaraldehyde, glycoaldehyde	Hemopure (HBOC-201)	↑ size, ↓ extravasation, ↑ half-life	NO scavenging leading to vasoconstriction, hypertension	Approved in South Africa; veterinary use in the US
Cross-linking	3,5-dibromosalicyl fumarate α–α cross-link at Lys99	DCLHb (HemAssist)	Stabilize tetramer, prevent renal clearance	Loss of cooperativity, hypertension, ↑ mortality	Phase III terminated, development stopped
Conjugation	Hb + antioxidant enzymes, PEGylation	PEG-Hb, enzyme-linked Hb	Antioxidant protection, ↑ size	Oxidative stress, inflammation, MI, death	Development stopped
Hybrids	Combined approaches (poly + crosslink, etc.)	Experimental only	Combine benefits	No clinical success to date	Experimental

Note: Table 1 is the author’s original compilation (own table). Arrows indicate the direction of change (↑ increase; ↓ decrease).

**Table 2:** HBOCs evaluated in clinical trials. Adapted from Harrington & Wollocko (2010), reproduced with permission from Springer (license on file) [28].

Product	Company	Description of Product-Source	Molecular Weight [kDa]
HemAssist	Baxter (Deerfield, IL, USA)	Diaspirin Cross-linked Human Hb	64
Hemopure	Biopure (Cambridge, MA, USA)	Glutaraldehyde cross-linked bovine Hb	Polymeric, 150
Hemolink	Hemosol (Mississauga, ON, Canada)	Raffinose cross-linked Human Hb	Polymeric, >120
PolyHeme	Northfield (Evanston, IL, USA)	Pyridoxilated, glutaraldehyde cross-linked human Hb	Polymeric, >120
Optro	Baxter (Deerfield, IL, USA) /Sommatogen (Boulder, CO, USA)	Recombinant human Hb, rHb1.1	64
Hemospan	Sangart (San Diego, CA, USA)	Pegylated human Hb	About 90

Note: Table 2 adapted from Harrington & Wollocko (2010) [28], reproduced with permission from Springer (license on file).

## Data Availability

No new data were created or analyzed in this study. Data sharing is not applicable to this article.
